# Correction: Diagnostic performance of eNose technology in detecting colorectal cancer recurrence: A prospective evaluation

**DOI:** 10.1371/journal.pone.0342238

**Published:** 2026-02-02

**Authors:** The PLOS One Staff

The sixth author’s initials appear incorrectly in the citation. The correct citation is: Schoenaker IJH, van Westreenen HL, Finnema EJ, Schrauwen R, Brohet RM, de Vos tot Nederveen Cappel, WH (2026) Diagnostic performance of eNose technology in detecting colorectal cancer recurrence: A prospective evaluation. PLoS One 21(1): e0340276. https://doi.org/10.1371/journal.pone.0340276

The publisher apologizes for the error.
