# Supplementary material for: Diagnostic performance of eNose technology in detecting colorectal cancer recurrence: A prospective evaluation
Source: PLoS One. 2026 Jan 7;21(1):e0340276. doi: 10.1371/journal.pone.0340276 (PMC12779126; doi:10.1371/journal.pone.0340276)
Supplement: S1 Table — (DOCX) [file pone.0340276.s001.docx]

## S1 Table. Patient, tumour and breath test characteristics for the training and test set

|  | **Total** | **Training set** | **Test set** | **P-value** |
| --- | --- | --- | --- | --- |
|  | n=406 | n=307 | n=99 |  |
| **Patient characteristics** |  |  |  |  |
| **Age** |  |  |  | 0.985^c^ |
| Mean ± SD | 68 (11) | 68 (11) | 68 (11) |  |
| **Gender** |  |  |  | 0.414^a^ |
| Female | 166 (41) | 129 (42) | 37 (37) |  |
| **BMI kg/m2** |  |  |  |  |
| Mean ± SD | 26,6 (4,5) | 26,6 (4.7) | 26,6 (3,8) | 0.908^c^ |
| **ASA** |  |  |  | 0.328^a^ |
| I | 106 (26) | 77 (25) | 29 (29) |  |
| II | 256 (63) | 193 (63) | 63 (63) |  |
| ≥ III | 44 (11) | 37 (12) | 7 (7) |  |
| **Comorbidity** |  |  |  | 0.752^a^ |
| Yes | 284 (70) | 216 (70) | 68 (69) |  |
| **Tumour characteristics** | **Total** | **Training set** | **Test set** | **P-value** |
| **Localization primary tumour** |  |  |  | 0.835^a^ |
| RCC | 167 (41) | 124 (40) | 43 (43) |  |
| LCC | 137 (34) | 104 (34) | 33 (33) |  |
| Rectal | 102 (25) | 79 (26) | 23 (23) |  |
| **MMR-status** |  |  |  | 0.620^a^ |
| MMRp (proficient | 247 (61) | 184 (60) | 63 (64) |  |
| MMRd (deficient) | 41 (10) | 30 (10) | 11 (11) |  |
| Missing | 118 (29) | 93 (30) | 25 (25) |  |
| **Neo-adjuvant therapy** |  |  |  | 0661^a^ |
| Yes | 80 (20) | 62 (20) | 18 (18) |  |
| **Tumour Stage **** |  |  |  | 0.606^a^ |
| I | 65 (16) | 52 (17) | 13 (13) |  |
| II | 126 (31) | 92 (30) | 34 (34) |  |
| III | 187 (46) | 140 (46) | 47 (47) |  |
| IV | 28 (7) | 23 (7) | 5 (5) |  |
| **Adjuvant therapy** |  |  |  | 0.662^a^ |
| Yes | 112 (28) | 83 (27) | 29 (29) |  |
| **Breath test characteristics** | **Total** | **Training set** | **Test set** | **P-value** |
| **Time after surgery (months)** |  |  |  | 0.189^d^ |
| Mean ± SD | 17 (14) | 16 (14) | 18 (13) |  |
| Median (IQR) | 11 (16) | 11 (15) | 13 (20) |  |
| **eNose device** |  |  |  | 0.775^a^ |
| Nr 40, older device | 324 (80) | 224 (79) | 80 (81) |  |
| Nr 13 , newer device | 82 (20) | 63 (21) | 19 (19) |  |
| **Current smoking** |  |  |  | 0.848^a^ |
| Yes | 31 (8) | 23 (7) | 8 (8) |  |
| **Diet** |  |  |  | 0.974^a^ |
| Yes | 29 (7) | 22 (7) | 7 (7) |  |
| **Last meal** |  |  |  | 0.671^a^ |
| < 3 hours | 144 (36) | 111 (36) | 33 (34) |  |
| >3 Hours | 258 (64) | 194 (64) | 64 (66) |  |
| **Alcohol < 24 hours**  **(missing 36)** |  |  |  | 0.798^a^ |
| Yes | 122 (33) | 91 (33) | 31 (34) |  |
| **Stoma** |  |  |  | 0.345^a^ |
| Yes | 58 (14) | 41 (13) | 17 (17) |  |
| **Medication** |  |  |  | 0.638^a^ |
| Yes | 271 (67) | 203 (66) | 68 (69) |  |
| **Supplements** |  |  |  | 0.850^a^ |
| Yes | 157 (39) | 120 (39) | 37 (38) |  |
| **CEA serum level ng/ml** |  |  |  | 0.123^a^ |
| <5 ng/ml | 343 (85) | 263 (87) | 80 (81) |  |
| ≥ 5 ng/ml | 58 (15) | 39 (13) | 19 (19) |  |

*Data are expressed as n (%) unless otherwise specified.*

*** in case of a ypTNM stage the cTNM classification is taken*

*ASA;American Society of Anesthesiologists; BMI; Body mass index; RCC; coecum to splenic flexure, LCC; splenic flexure to rectum, included recto-sigmoid, RC; rectal cancer; MMR; mismatch repair*

*^a^ Pearson Chi-Square test*, *^b^ Fisher’s Exact Test, ^c^ Independent-Samples T-test*, *^d^ Mann-Whitney U test*
