# Supplementary material for: Diagnostic performance of eNose technology in detecting colorectal cancer recurrence: A prospective evaluation
Source: PLoS One. 2026 Jan 7;21(1):e0340276. doi: 10.1371/journal.pone.0340276 (PMC12779126; doi:10.1371/journal.pone.0340276)
Supplement: S2 Table — (DOCX) [file pone.0340276.s003.docx]

## S3 Table. Patient, tumour and breath test characteristics for the correctly and incorrectly predicted patients in the test set

|  | **Total** | **Correctly predicted** | **Incorrectly predicted** | **Missing** | **P-value** |
| --- | --- | --- | --- | --- | --- |
|  | n=99 | n= 50 | n= 49 |  |  |
| **Patient characteristics** |  |  |  |  |  |
| **Age** |  |  |  | 0 | 0.511^c^ |
| Mean ± SD | 68 (11) | 69 (11) | 67 (10) |  |  |
| **Gender** |  |  |  | 0 | 0.896^a^ |
| Female | 37 (37) | 19 (38) | 18 (37) |  |  |
| **BMI kg/m2** |  |  |  |  |  |
| Mean ± SD | 26,7 (3,8) | 26,7 (3,6) | 26,7 (4,0) | 0 | 0.923^c^ |
| **ASA** |  |  |  | 0 | 0.585^b^ |
| I | 29 (29) | 15 (30) | 14 (29) |  |  |
| II | 63 (64) | 33 (66) | 30 (61) |  |  |
| ≥ III | 7 (7) | 2 (4) | 5 (10) |  |  |
| **Comorbidity n (%)** |  |  |  | 0 | 0.560^a^ |
| Yes | 68 (69) | 33 (66) | 35 (71) |  |  |
| **Tumour characteristics** | **Total** | **Correctly predicted** | **Incorrectly predicted** | **Missing** | **P-value** |
| **Localization primary tumour** |  |  |  | 0 | 0.733^a^ |
| RCC | 43 (43) | 20 (40) | 23 (47) |  |  |
| LCC | 33 (33) | 17 (34) | 16 (33) |  |  |
| Rectal | 23 (23) | 13 (26) | 10 (20) |  |  |
| **MMR-status** |  |  |  | 0 | 0.259^a^ |
| MMRp (proficient | 63 (63) | 34 (68) | 29 (59) |  |  |
| MMRd (deficient) | 11 (11) | 3 (6) | 8 (16) |  |  |
| Missing | 25 (25) | 13 (26) | 12 (25) |  |  |
| **Neo-adjuvant therapy** |  |  |  | 0 | 0.570 ^a^ |
| Yes | 18 (18) | 8 (16) | 10 (20) |  |  |
| **Tumour Stage**** |  |  |  | 0 | 0.962^b^ |
| I | 13 (13) | 7 (14) | 6 (12) |  |  |
| II | 34 (34) | 18 (36) | 16 (33) |  |  |
| III | 47 (47) | 23 (46) | 24 (49) |  |  |
| IV | 5 (5) | 2 (4) | 3 (6) |  |  |
| **Adjuvant therapy** |  |  |  | 0 | 0.467^a^ |
| Yes | 29 (29) | 13 (26) | 16 (33) |  |  |
| **Recurrence characteristics** | **Total** | **Correctly predicted** | **Incorrectly predicted** | **Missing** |  |
| **Distant metastases** | 22 | 10 | 12 |  |  |
| **Locoregional recurrence** | 3 | 3 | 0 |  |  |
| One-organ | 18 (72) | 8(61) | 10 (83) | 0 | 0.378^b^ |
| *Liver* | 13 | 6 | 7 |  |  |
| *Pulmonal* | 8 | 2 | 6 |  |  |
| *Peritoneal* | 3 | 3 | 0 |  |  |
| *Lymphatic* | 4 | 3 | 1 |  |  |
| *Other* | 2 | 1 | 1 |  |  |
| **Breath test characteristics** | **Total** | **Correctly predicted** | **Incorrectly predicted** | **Missing** | **P-value** |
| **Time after surgery (months)** |  |  |  |  |  |
| Mean ± SD | 18 (4) | 18 (11) | 18 (15) | 0 | 0.277^d^ |
| Median (IQR) | 13 (20) | 16 (20) | 8 (22) |  |  |
| **eNose device** |  |  |  | 0 | 0.220^a^ |
| Nr 40, older device | 80 (81) | 38 (76) | 42 (86) |  |  |
| Nr 13, newer device | 19 (19) | 12 (24) | 7 (14) |  |  |
| **Current smoking** |  |  |  | 0 | 0.487^b^ |
| Yes | 8 (8) | 3 (6) | 5 (10) |  |  |
| **Diet** |  |  |  | 0 | 0.269^b^ |
| Yes | 7 (7) | 2 (4) | 5 (10) |  |  |
| **Last meal** |  |  |  | 2 | 0.474^a^ |
| < 3 hours | 33 (34) | 15 (31) | 18 (37) |  |  |
| >3 Hours | 64 (66) | 34 (69) | 30 (63) |  |  |
| **Alcohol < 24 hours** |  |  |  | 8 | 0.141^a^ |
| Yes | 31 (34) | 19 (41) | 12 (27) |  |  |
| **Stoma** |  |  |  | 0 | 0.825^a^ |
| Yes | 17 (17) | 9 (18) | 8 (16) |  |  |
| **Medication** |  |  |  | 0 | 0.776^a^ |
| Yes | 68 (69) | 35 (70) | 33 (67) |  |  |
| **Supplements** |  |  |  | 2 | **0.017^a^** |
| Yes | 37 (38) | 13 (27) | 24 (50) |  |  |
| **CEA serum level ng/ml** |  |  |  | 0 | 0.837^a^ |
| < 5 ng/ml | 80 (81) | 40 (80) | 40 (82) |  |  |
| ≥ 5 ng/ml | 19 (19) | 10 (20) | 19 (18) |  |  |

*Data are expressed as n (%) unless otherwise specified.*

*** in case of a ypTNM stage the cTNM classification is taken*

*ASA;American Society of Anesthesiologists; BMI; Body mass index; RCC; coecum to splenic flexure, LCC; splenic flexure to rectum, included recto-sigmoid, RC; rectal cancer; MMR; mismatch repair*

*^a^ Pearson Chi-Square test*, *^b^ Fisher’s Exact Test, ^c^ Independent-Samples T-test*, *^d^ Mann-Whitney U test*
